# Association between immune-related adverse events and recurrence dynamics under adjuvant anti–PD-1 therapy in resected melanoma

**DOI:** 10.3389/fonc.2025.1671315

**Published:** 2025-11-07

**Authors:** Gökhan Şahin, Caner Acar, Haydar Çağatay Yüksel, Salih Tünbekici, Pınar Açar, Banu Yaman, Burçak Karaca

**Affiliations:** 1 Department of Medical Oncology, Ege University Medical Faculty, Izmir, Türkiye; 2 Department of Pathology, Ege University Medical Faculty, Izmir, Türkiye

**Keywords:** melanoma, immune-related adverse events, anti–PD-1 therapy, recurrence-free survival, adjuvant immunotherapy, cutaneous irAEs

## Abstract

**Introduction:**

Adjuvant anti–PD-1 therapy has significantly improved recurrence-free survival (RFS) in patients with resected high-risk melanoma, yet a substantial proportion still relapse. Identifying predictors of recurrence remains a clinical priority.

**Methods:**

We retrospectively analyzed 84 patients with resected stage IIB–IV cutaneous melanoma who received adjuvant nivolumab or pembrolizumab. Clinicopathologic factors and immune-related adverse events (irAEs) occurring within the first 6 months of therapy were evaluated for their association with recurrence timing and RFS. Kaplan–Meier estimates, Cox regression models, and 3- and 6-month landmark analyses were used.

**Results:**

After a median follow-up of 30.7 months, 48.8% of patients experienced recurrence, including 25 patients who recurred during treatment. Recurrence during therapy was associated with advanced stage, greater Breslow thickness, and acral subtype. Cutaneous irAEs were significantly less frequent in patients who recurred during treatment (6.7% vs. 36.2%, p=0.010). In univariate Cox regression, cutaneous irAEs were associated with improved RFS (hazard ratio [HR]: 0.18; 95% CI: 0.04–0.73; p=0.017). This association remained significant in multivariate analysis (HR: 0.07; 95% CI: 0.01–0.56; p=0.011), independent of other clinicopathologic variables. Landmark analyses at 3 and 6 months confirmed the prognostic relevance of early cutaneous irAEs.

**Conclusions:**

Cutaneous irAEs within 6 months of initiating adjuvant PD-1 blockade are independently associated with reduced recurrence risk and prolonged RFS. Early emergence of these events may serve as a dynamic marker of effective anti-tumor immunity in patients with resected high-risk melanoma.

## 1 Introduction

Stage IIB/IIC and stage III melanomas are considered high-risk due to their substantial potential for recurrence and metastasis. Historically, survival rates have shown a marked decline with increasing stage, as reflected by 10-year survival rates of approximately 82% for stage IIB, 24% for stage IIID, and below 10% for metastatic disease (1, 2). With the advancements in systemic therapies, including immunotherapy and targeted therapies, the survival outcomes have improved dramatically (3).

The success of immunotherapies in the metastatic setting prompted researchers to explore their role as adjuvant treatments for high-risk melanoma. Ipilimumab was the first to demonstrate improved recurrence-free survival (RFS) and overall survival (OS) in stage III patients (4). Then Programed cell death protein 1 (PD-1) inhibitors pembrolizumab and nivolumab were introduced. The CheckMate 238 trial showed that nivolumab prolonged RFS compared to ipilimumab in patients with resected stage IIIB, IIIC or IV melanoma (5). Similarly, EORTC 1325/KEYNOTE 054 and KEYNOTE 716 trials showed that pembrolizumab prolonged RFS in stage III and IIB/IIC respectively compared to placebo (6, 7). More recently, the CheckMate 76K trial confirmed the efficacy of adjuvant nivolumab in resected stage IIB and IIC melanoma, showing a significant RFS benefit over placebo (8). So adjuvant anti-PD-1 therapy (nivolumab and pembrolizumab) is the standard of care for high risk resected melanoma.

Despite these advances, approximately 60% of tumors exhibit primary resistance to anti–PD-1 therapy, and 35% of initial responders eventually develop acquired resistance (9). Following adjuvant anti–PD-1 therapy, 25–30% of patients experience recurrence within 1 year, which is associated with worse survival outcomes (10, 11). Accurately identifying which patients are more likely to relapse during or after adjuvant treatment remains a major challenge, highlighting the need for reliable predictive biomarkers and refined risk stratification tools.

Recent efforts have focused on circulating tumor DNA (ctDNA), immune profiling, and histopathological features to improve prognostic modeling in this context. For instance, a meta-analysis confirmed that ctDNA positivity was strongly associated with worse progression-free and overall survival in patients treated with immune checkpoint inhibitors (12). Other proposed biomarkers include tumor mutational burden (TMB), MHC expression, TIL density, and composite immune-related gene signatures (13).

Alongside these molecular tools, immune-related adverse events (irAEs) have emerged as accessible clinical markers of treatment efficacy. In metastatic melanoma, several large-scale meta-analyses have consistently demonstrated an association between irAEs and improved overall and progression-free survival (14, 15). However, in the adjuvant setting, this relationship has been less consistently observed, and the available evidence is limited to a few key trials. Among these, the EORTC 1325/KEYNOTE-054 trial demonstrated that the development of irAEs during pembrolizumab treatment was associated with improved RFS, suggesting a potential protective effect (16). Conversely, a similar analysis of the CheckMate 238 trial, which evaluated adjuvant nivolumab, did not find a similar association, indicating potential differences in irAEs’ predictive value across treatment regimens (17). These findings underscore the need for further research to clarify the role of irAEs in recurrence dynamics and their potential as stratification tools in adjuvant therapy.

Given the complexities of predicting outcomes in high-risk melanoma treated with adjuvant anti–PD-1 therapy, our study aimed to explore both recurrence under therapy and RFS in relation to a set of clinical and pathological variables. Specifically, we investigated the prognostic value of irAEs occurring within the first 6 months of treatment and their association with both recurrence dynamics and RFS in patients receiving adjuvant nivolumab or pembrolizumab.

## 2 Methods

### 2.1 Study design and ethical approval

This is a retrospective, single-center study conducted at the Department of Medical Oncology, Ege University. The study population consisted of adult patients (≥18 years) with histologically confirmed cutaneous melanoma who received adjuvant anti–PD-1 therapy (nivolumab or pembrolizumab) between September 3, 2015, and August 29, 2024. Patients with mucosal or uveal melanoma or concurrent malignancies were excluded. The study protocol was approved by the Institutional Review Board of Ege University Hospital.

### 2.2 Study population, data collection and variables

We identified adult patients (≥18 years) with cutaneous melanoma who underwent complete resection for stage IIB, IIC, III, or IV disease according to the AJCC 8th edition, and subsequently received adjuvant anti–PD-1 monotherapy (nivolumab or pembrolizumab). Patients with mucosal melanoma, uveal melanoma, or concurrent malignancies were excluded. After applying these criteria, a total of 84 patients were included in the final analysis.

We evaluated patient demographics (e.g., age, gender) and tumor characteristics (primary tumor site, lymph node metastasis, ulceration, Breslow thickness, mitotic rate, and BRAF mutation status). Additionally, we assessed the presence of irAEs, their timing, and the recurrence status during follow-up.

To assess recurrence dynamics more precisely, disease stages were categorized into three prognostic risk groups based on recurrence rates and RFS: Group 1: Stage IIB and IIIA, Group 2: Stage IIC and IIIB, Group 3: Stage IIIC–IIID–IV (18–20). Adjuvant anti–PD-1 therapy was administered for a planned duration of 12 months. Nivolumab was given at a dose of 240 mg every 2 weeks or 3 mg/kg every 2 weeks, and pembrolizumab was administered at 200 mg every 3 weeks, in accordance with standard clinical practice.

### 2.3 Definition of recurrence-free survival and adverse event monitoring

RFS was defined as the time from the date of complete surgical resection to the date of first documented recurrence (local, regional, or distant) or death from any cause, whichever occurred first. IrAEs were assessed and graded according to the Common Terminology Criteria for Adverse Events (CTCAE) version 5.0. (21). Cutaneous irAEs were primarily diagnosed by the treating oncologist based on clinical presentation. Dermatology consultation was obtained in selected cases with atypical or persistent lesions. Skin biopsy or imaging was not routinely performed, but used when clinically indicated.

To minimize bias arising from differences in follow-up duration and treatment exposure between patients with and without irAEs, we focused on irAEs occurring within the first 6 months of therapy, as the majority of irAEs are known to develop during this period (22, 23). Cutaneous and endocrine irAEs were analyzed separately given their relatively higher frequency in the cohort, while less common irAEs (e.g., gastrointestinal, hepatic, rheumatic, pancreatic) were grouped as “other irAEs” due to small numbers.

### 2.4 Statistical analysis

All statistical analyses were performed using SPSS version 28.0 (IBM Corp., Armonk, NY) and Jamovi version 2.3. Categorical variables were summarized as frequencies and percentages; continuous variables were reported as medians with interquartile ranges (IQRs). Comparisons between categorical variables were conducted using the chi-square or Fisher’s exact test, as appropriate. A two-sided p-value < 0.05 was considered statistically significant.

Additionally, patients were categorized into two groups based on recurrence status during anti–PD-1 therapy (12months): those who developed recurrence during treatment (“Recurred” group) and those who completed therapy without recurrence (“No Recurrence” group). Clinicopathologic characteristics and immune-related adverse events were compared between these groups using the chi-square or Fisher’s exact test, as appropriate.

RFS was estimated using the Kaplan–Meier method, and group differences were assessed with the log-rank test. Median follow-up time was calculated using the reverse Kaplan–Meier method, censoring events and deaths to avoid bias. To explore the association between variables and RFS, univariate Cox regression analyses were performed. Variables with p < 0.10 in univariate analysis or those deemed clinically relevant were included in the multivariable Cox regression model. Covariates included established prognostic indicators such as stage (AJCC 8th edition), Breslow thickness, lymph node involvement, ulceration status, mitotic rate, and BRAF mutation status. Hazard ratios (HRs) and 95% confidence intervals (CIs) were reported.

To address potential time-dependent bias and immortal time bias, landmark analyses were performed at 3 and 6 months. Patients who experienced recurrence before the respective landmark time points were excluded. Among the remaining patients, RFS was calculated from the landmark time onward. Patients were categorized based on whether they had experienced cutaneous irAE prior to the landmark. Comparisons of RFS between patients with and without cutaneous irAEs were performed using Kaplan–Meier survival analysis and the log-rank test.

## 3 Results

A total of 102 patients with melanoma who received adjuvant anti–PD-1 monotherapy were initially screened. After excluding patients with mucosal melanoma, uveal melanoma, or other concurrent malignancies, 84 patients with cutaneous melanoma aged 18 years or older were included in the final analysis.

The median follow-up duration was 30.7 months (95% CI, 25.6–35.8). Among the 84 patients, 25 (29.8%) experienced recurrence during anti–PD-1 therapy, while the remaining 59 (70.2%) completed treatment without recurrence. Of these 59 patients, 16 later developed recurrence during follow-up. In total, 41 patients (48.8%) experienced disease recurrence by the time of data cut-off. 17 patients (20.2%) had died. The majority of patients were under the age of 65 years (65.5%), and 56% were male. Most tumors were of primary origin (77.1%), while 22.9% represented recurrent cases. Among those with recurrent tumors, none had received prior adjuvant systemic therapy after initial surgery. Stage IIIC disease was the most common at diagnosis (46.4%), followed by stage IIIB (13.1%), and stage IV (10.7%). The primary tumor was most frequently located on the extremities (38.5%), followed by the trunk (32.3%) and head/neck region (20%).

Breslow thickness exceeded 4 mm in 48.8% of patients, and ulceration was present in 61.9%. Lymph node involvement was observed in 70.2% of patients, with 34.5% having ≥2 positive nodes. Histologically, the most frequent subtypes were nodular melanoma (23.8%) and superficial spreading melanoma (23.8%), followed by acral lentiginous melanoma (15.5%). BRAF mutation was present in 26.2% of patients, absent in 65.6%, and unknown in 8.3%. Mitotic index was reported as >10/mm² in 35.7%, ≤10/mm² in 36.9%, and not evaluated in 27.4% of patients.

Regarding the type of anti–PD-1 agent received, 64 patients (76.2%) were treated with nivolumab, and 20 patients (23.8%) received pembrolizumab. Baseline characteristics are summarized in **Table 1**.

**Table 1:** Baseline demographic, clinical, pathological, and treatment characteristics of the study cohort.

	n (%)
Age	
<65.0 years	55(65.5)
≥65.0 years	29(34.5)
Gender	
Male	47(56)
Female	37(44)
Tumor origin	
Primary	65(77.4)
Recurrence	19(22.6)
Stage	
IIB	9(10.7)
IIC	6(7.1)
IIIA	6(7.1)
IIIB	11(13.1)
IIIC	39(46.4)
IIID IV	4(4.8) 9(10.7)
Breslow thickness	
>1-2 mm	8(9.5)
>2-4 mm	16(19)
>4mm	41(48.8)
NE	19(22.6)
Tumor ulceration	
No	13(15.5)
Yes	52(61.9)
NE	19(22.6)
Lymph node involvement	
0	16(19)
1	30(35.7)
≥2	29 (34.5)
NE	9(10.7)
Melanoma Location	
Head and neck	13(20)
Trunk	21(32.3)
Extremity	25(38.5)
NE	6(9.2)
Histological type	
Nodular	20(23.8)
Superficial spreading	20(23.8)
Acral lentiginous melanoma	13(15.5)
NE	31(36.9)
Mitosis	
1-10	31(36.9)
>10	30(35.7)
NE	23(27.4)
BRAF mutation	
Present	22(26.2)
Absent	55(65.6)
NE	7(8.3)
Anti-PD1 Type	
Pembrolizumab	20(23.8)
Nivolumab	64(76.2)
Recur during on adjuvant treatment	
No	59(70.2)
Yes	25(29.8)
Any irAE	34(40.5)
irAE grade	
Grade 1	12(35.3)
Grade 2	16(47.1)
Grade 3	3(8.8)
Grade 4	3(8.8)
Endocrine irAE	9(10.7)
Cutaneous irAE	15(17.9)
Other irAE	10(11.9)
Grade 3-4 irAE	6(7.1)
Steroid use due to irAE	9(10.7)
Recurrence during follow-up	
Yes	41(48.8)
No	43(51.2)
Recurrence Type	
Local recurrence	8(19.5)
Distant recurrence	33(80.5)
Treatment after recurrence	
Local treatment (Surgery/RT)	2(4.9)
Local treatment + Systemic treatment	18(43.9)
Systemic treatment	21(51.2)
First-line treatment after recurrence	
Continuation of anti-PD1	1 (39)
Nivolumab + Ipilimumab	14(34.1)
BRAF/MEK inhibitors	6(14.6)
Chemotherapy	3(7.3)
Mortality	17(20.2)

NE, Not evaluated; LN, Lymph node; PD-1, Programmed cell death protein 1; irAE, Immune-related adverse event; RT, Radiotherapy; RFS, Recurrence-free survival; BRAF, B-Raf proto-oncogene; MEK, Mitogen-activated protein kinase.

### 3.1 Immune-related adverse events within the first 6 months

A total of 34 patients (40.5%) developed irAEs within the first 6 months of anti–PD-1 therapy. Cutaneous irAEs were the most common, observed in 15 patients (44.1% of those with irAEs), followed by endocrine irAEs in 9 patients (26.5%). All cutaneous irAEs were Grade 1–2.

Other irAE subtypes were less common, including hepatic (n=3), rheumatic (n=2), gastrointestinal (n=4), and pancreatic irAEs (n=1). Notably, two patients developed Grade 3–4 colitis, and one experienced Grade 4 pancreatitis. 9 patients (10.7%) required systemic corticosteroids for irAE management, most commonly in the setting of gastrointestinal or hepatic toxicity. A full breakdown of irAEs by type and severity grade is presented in **Table 2**.

**Table 2:** Rates of irAEs within 6 months ordered by frequency and severity grade.

irAE	Any Grade n(%)	Grade 1 n(%)	Grade 2 n(%)	Grade 3 n(%)	Grade 4 n(%)
Cutaneous	15 (44.1%) 6	4	2		
Vitiligo Rash Pruritis	6 3	3	3 3		
Endocrine	9(26.5%)				
Hypothyroidism Hyperthyroidism Hypophysitis	3 5 1	2 2	1 3	1	
Hepatic	3(8.8%)	1	2		
Rheumatic (Artralgia)	2(5.9%)	1	1		
Gastrointestinal (Colitis)	4(11.7%)			2	2
Pancreatitis	1(2.9%)				1

### 3.2 Recurrence during anti-PD1 treatment: associated factors

Among the 84 patients who received adjuvant anti–PD-1 therapy, 25 (29.8%) experienced disease recurrence during treatment, while 59 (70.2%) completed the planned therapy without recurrence. Clinicopathological characteristics were compared between these two groups to identify factors associated with recurrence during active treatment.

Patients who recurred during treatment more frequently exhibited high-risk disease features, including stage IIIC–IV melanoma (38.5% vs. 51.9%, p = 0.016) and greater Breslow thickness (>4 mm: 32.3% vs. 51.3%, p = 0.021). Acral melanoma subtype was also more prevalent in this group (53.8% vs. 25.4%, p = 0.039). Although not statistically significant, a higher rate of lymph node involvement ≥2 was observed among patients who recurred during treatment (48.3% vs. 25.4%, p = 0.053).

The presence of cutaneous irAEs was significantly less frequent in patients with recurrence during therapy compared to those who recurred after completing therapy (6.7% vs. 36.2%, p=0.010). Endocrine irAEs were also numerically lower in this group (55.6% vs. 26.7%), but did not reach statistical significance (p=0.073). Other baseline characteristics, including age, gender, tumor ulceration, BRAF mutation status, mitotic index, melanoma location, anti–PD-1 type and steroid use, did not differ significantly between the two groups (**Table 3**).

**Table 3:** Factors associated with recurrence during anti–PD-1 therapy.

	No recurrence during anti-PD-1 n=59	Recurred during anti-PD1 n=25	P value
Age			0.853
<65.0 years	39(70.9)	16(29.1)	
≥65.0 years	20(69.0)	9(31.0)	
Gender			0.635
Male	25(67.7)	12(32.4)	
Female	34(72.3)	13(27.7)	
Tumor origin			0.327
Primary	43(67.2)	21(32.8)	
Recurrence	15(78.9)	4(21.1)	
Stage			0.016
IIB and IIIA	15(100)	0(0)	
IIC and IIIB	12(70.6)	5(29.4)	
IIIC-IIID-IV	32(61.5)	20(38.5)	
Breslow thickness			0.021
1-2 mm	8(100)	0(0)	
2-4 mm	13(81.3)	3(18.8)	
>4mm	23(56.1)	21(32.3)	
Tumor ulceration			0.894
Yes	35(67.3)	17(32.7)	
No	9(69.2)	4(30.8)	
Lymph node involvement			0.053
0	13(81.3)	3(18.8)	
1	23(76.7)	7(23.3)	
≥2	15(51.7)	14(48.3)	
Melanoma location			0.493
Head and neck	13(81.3)	3(18.8)	
Trunk	18(69.2)	8(30.8)	
Extremity	22(64.7)	12(35.3)	
Melanoma subtype			0.039
Non-acral cutaneous	53(74.6)	18(25.4)	
Acral	6(46.2)	7(53.8)	
Mitosis			0.717
1-10	21(67.7)	10(32.3)	
10	19(63.3)	11(36.7)	
BRAF mutation			0.813
Present	15(68.2)	7(31.8)	
Absent	39(70.9)	16(29.1)	
Anti-PD1 Type			0.594
Pembrolizumab	15(75.0)	5(25.0)	
Nivolumab	44(68.8)	20(31.3)	
Any irAE			0.129
Yes	27(79.4)	7(20.6)	
No	32(64.0)	18(36.0)	
Endocrine irAE			0.073
Yes	4(44.4)	5(55.6)	
No	55(73.3)	20(26.7)	
Cutaneous irAE			0.010
Yes	14(93.3 )	1(6.7)	
No	44(63.8)	25(36.2)	
Other irAE			0.472
Yes	8(80.0)	2(20.0)	
No	51(68.9)	23(31.1)	
Steroid use due to irAE			0.103
Yes	8(14.8)	1(3.3)	
No	46(85.2)	29(96.7)	
Grade 3-4 irAE			0.467
Yes	5(83.3)	1(16.7)	
No	53(69.2)	24(30.8)	

irAE, Immune-related adverse event; PD-1, Programmed death-1; NE, Not evaluated; P values less than 0.05 are shown in bold.

### 3.3 Risk factors for RFS (univariate and multivariate Cox regression analysis)

The median RFS for the entire cohort was 26.9 months, and the 1-year RFS rate was 70.2%. In univariate Cox regression analysis, the occurrence of cutaneous irAEs within the first 6 months of treatment was significantly associated with improved RFS (HR: 0.18, 95% CI: 0.04–0.73, p=0.017). Additionally, patients with acral melanoma subtype (HR: 1.95, 95% CI: 0.93–4.10, p=0.077) and those with ≥2 involved lymph nodes (HR: 2.54, 95% CI: 1.01–6.32, p=0.051) showed a trend toward shorter RFS.

In multivariate Cox regression analysis, the presence of cutaneous irAEs within the first 6 months remained independently associated with longer RFS (HR: 0.07, 95% CI: 0.01–0.56, p=0.011). Other clinical variables, including age, sex, tumor origin, disease stage, Breslow thickness, lymph node involvement, melanoma subtype, mitotic index, anti–PD-1 type and BRAF mutation status, were not significantly associated with RFS. The complete results of univariate and multivariate analyses are summarized in **Table 4**.

**Table 4:** Factors associated with RFS (univariate and multivariate Cox regression analysis).

	HR	Univariate		Multivariate	P value
		95% CI	P value	HR (95% CI)	
Age			0.679		
<65.0 years	1				
≥65.0 years	1.15	0.60-2.17			
Gender			0.125		
Male	1				
Female	1.62	0.87-3.00			
Tumor origin			0.140		
Primary	1				
Recurrence	0.53	0.23-1.23			
Stage			0.109		0.505
IIB and IIIA	1				
IIC and IIIB	2.19	0.67-7.16		0.26(0.07-9.87)	
IIIC-IIID-IV	2.72	0.95-7.79		1.07(0.19-6.13)	
Breslow thickness			0.113		0.386
>1-2 mm	1				
>2-4 mm	0.74	0.21-2.63		4.72(0.40-55.9)	
>4mm	1.74	0.60-5.04		9.93(0.38-262.1)	
Tumor ulceration			0.714		0.169
No	1				
Yes	1.18	0.49-2.86		2.53(0.67-9.47)	
Lymph node involvement			0.051		0.080
0	1				
1	1.26	0.48-3.34		6.33(0.35-114.8)	
≥2	2.54	1.01-6.32		16.33(0.69-388.2)	
Melanoma Location			0.378		
Head and neck	1				
Trunk	1.80	0.69-4.68			
Extremity	1.79	0.71-4.51			
Melanoma subtype			**0.077**		0.413
Non-acral cutaneous	1				
Acral	1.95	0.93-4.10		1.54(0.54-4.40)	
Mitosis			0.134		0.257
1-10	1				
10	1.74	0.85-3.49		1.93(0.62-6.05)	
BRAF mutation			0.288		0.654
Absent	1				
Present	1.44	0.73-2.82		0.78(0.27-2.27)	
Anti-PD1 Type			0.190		
Pembrolizumab	1				
Nivolumab	1.73	0.72-4.13			
Presence of irAE			0.125		
No	1				
Yes	0.60	0.32-1.15			
Steroid use due to irAE			0633		
No	1				
Yes	0.748	0.22-2.46			
Endocrine irAE			0.267		
No	1				
Yes	1.64	0.69-3.90			
Cutaneous irAE			0.017		0.011
No	1				
Yes	0.18	0.04-0.73		0.07(0.01-0.56)	
Other irAE			0.922		
No	1.04				
Yes	1	0.44-2.49			
Grade 3-4 irAE			0.596		
No	1				
Yes	1.32	0.47-3.72			

irAE, Immune-related adverse event; PD-1, Programmed death-1; NE, Not evaluated; P values less than 0.05 are shown in bold.

**Figure 1:**
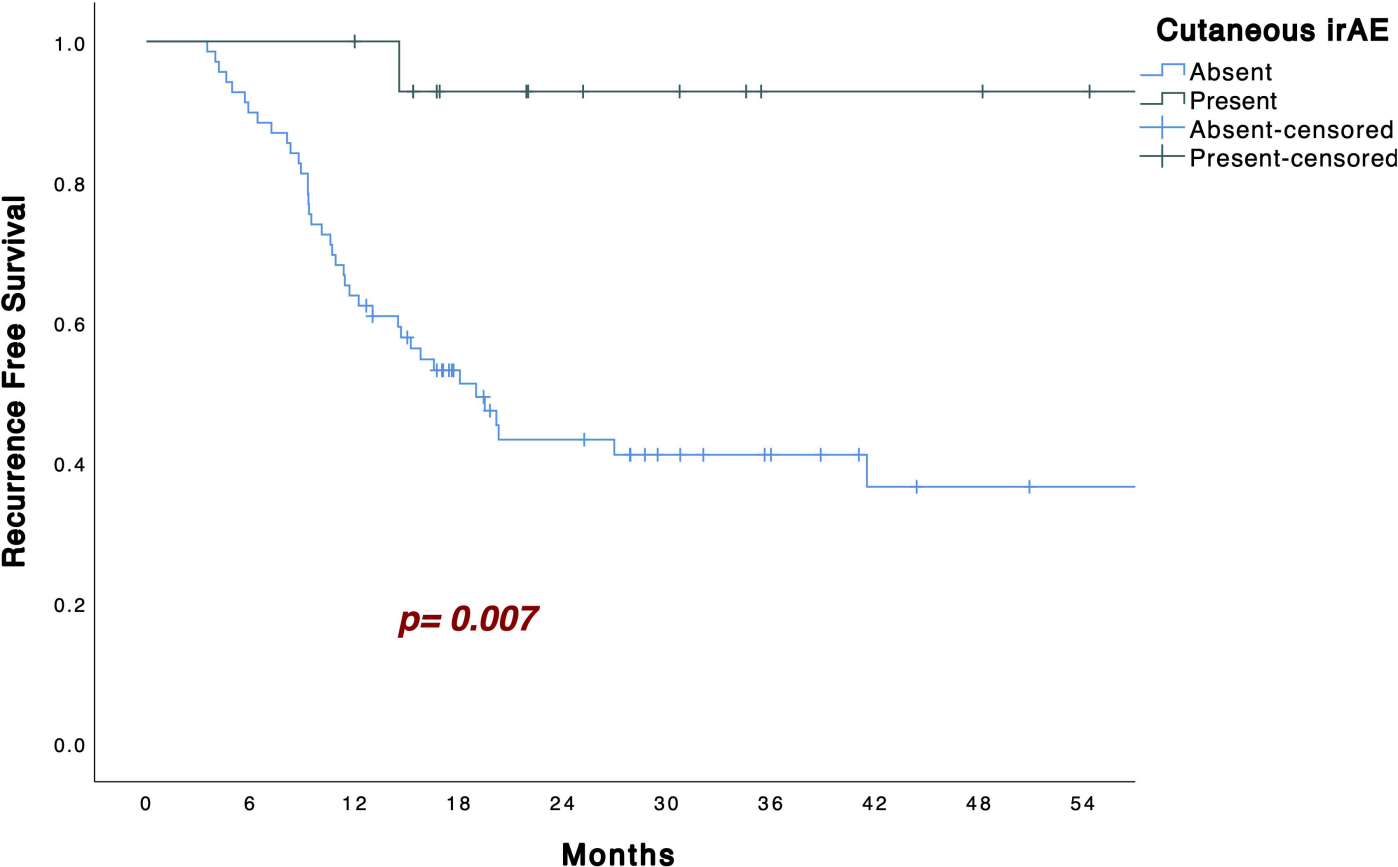
Kaplan–Meier curves for recurrence-free survival in patients with or without cutaneous irAEs.

**Figure 2:**
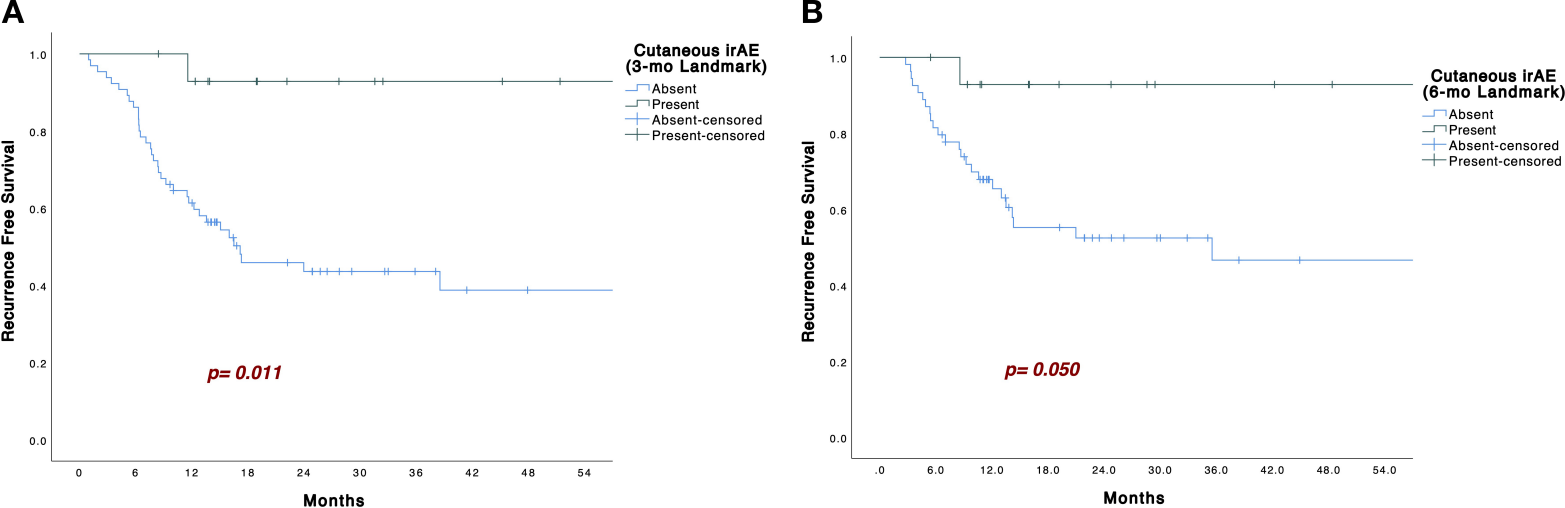
**(A)**. Kaplan–Meier curves with 3 months landmark analysis for recurrence-free survival in patients with or without cutaneous irAEs. **(B)**. Kaplan–Meier curves with 6 months landmark analysis for recurrence-free survival in patients with or without cutaneous irAEs.

Kaplan–Meier analysis showed that patients who developed cutaneous irAEs had significantly longer RFS than those who did not (p = 0.007; **Figure 1**). In the 3−month landmark analysis, four patients had relapsed before day 90; none of these four had developed cutaneous irAEs. By day 90, eight patients had developed cutaneous irAEs, and the association with longer RFS remained significant (p = 0.011; **Figure 2A**). In the 6−month landmark analysis, 15 patients had relapsed before day 180; again, none of these early−relapsing patients had developed cutaneous irAEs, and the protective association persisted among those still at risk (p = 0.050; **Figure 2B**).

### 3.4 Additional analyses of cutaneous irAEs

Given the central role of cutaneous irAEs in the prognostic stratification of patients receiving adjuvant anti–PD-1 therapy, additional analyses were performed to ensure that their association with RFS was not confounded by baseline clinicopathological features.

As shown in **Supplementary Table S1**, there were no statistically significant differences in key prognostic variables — including age, sex, disease stage, Breslow thickness, ulceration status, lymph node involvement, melanoma subtype, mitotic index, BRAF mutation status, or anti–PD-1 agent — between patients with and without cutaneous irAEs. These findings support the robustness of the observed association between cutaneous irAEs and improved RFS.

In addition, **Supplementary Table S2** presents detailed clinical, pathological, and outcome data for the 15 patients who developed cutaneous irAEs. Most of these events were low grade (Grade 1–2). Only two patients experienced recurrence during follow-up, further underscoring the potential protective effect of early-onset cutaneous irAEs.

## 4 Discussion

The advent of adjuvant immunotherapy has dramatically improved outcomes in high-risk melanoma. Multiple phase III trials have established PD-1 inhibitors (nivolumab or pembrolizumab) as standard of care in resected stage IIB–IV disease. For example, adjuvant nivolumab significantly prolonged RFS compared to ipilimumab in CheckMate 238, and pembrolizumab improved RFS versus placebo in both stage III (EORTC 1325/KEYNOTE-054) and stage IIB/IIC (KEYNOTE-716) trials (5–7). More recently, the CheckMate 76K trial confirmed a RFS benefit with nivolumab in stage IIB/IIC melanoma (8). Despite these advances, relapse rates remain substantial – approximately 25–30% of patients on adjuvant anti–PD-1 experience recurrence within the first year (6). Consistently, in our cohort, the 12-month RFS rate was 70.2%, indicating that nearly one-third of patients recurred within the first year of treatment. Given this early relapse pattern, we aimed to explore whether certain clinicopathologic or immunologic features were associated with recurrence during active therapy versus after its discontinuation.

Multiple baseline patient and tumor characteristics were identified as risk factors for melanoma recurrence during adjuvant immunotherapy. Our findings implicate advanced disease stage, greater tumor depth (Breslow thickness), regional lymph node involvement, and acral melanoma subtype as variables associated with a higher likelihood of recurrence during anti-PD-1 treatment. Conversely, the occurrence of cutaneous irAEs within the first 6 months was significantly less common among patients who experienced recurrence during active treatment (6.7% vs. 36.2%, p=0.010), suggesting a protective immune response against early relapse. For example, patients with more advanced stage (particularly stage III disease with nodal metastases) face relapse rates exceeding 50% despite adjuvant therapy, underscoring the impact of nodal involvement (24). Even in the absence of nodal disease, high-risk primaries defined by deep Breslow thickness have a substantial recurrence risk – Stage IIB/IIC melanomas (thickness >4 mm and/or ulceration) exhibit approximately 44–67% 10-year recurrence rates (25). Indeed, Breslow thickness has long been recognized as one of the most predictive features for melanoma recurrence (26). Our analysis also demonstrated that melanoma subtype significantly influences recurrence patterns during adjuvant anti–PD-1 therapy. Specifically, patients with acral melanoma showed a markedly higher rate of recurrence while receiving treatment compared to those with non-acral cutaneous melanoma (53.8% vs. 25.4%, p = 0.039). This observation is consistent with recent findings from Yusuke et al., who reported a higher risk of recurrence during PD-1 therapy in acral melanoma (27). Taken together, these examples from the literature reinforce that advanced stage (with nodal spread), deeper primary tumor thickness, and acral primary site are robust predictors of recurrence, corroborating our findings in patients who relapsed during anti–PD-1 treatment. Each of these factors likely reflects a higher baseline tumor burden or biologic aggressiveness that can overcome even effective therapies, leading to early relapse despite adjuvant treatment.

Previous studies suggest that irAEs may reflect the activation of immune pathways targeting both normal and tumor tissues, thereby enhancing anti-tumor efficacy and improving clinical outcomes (28, 29). This correlation has been demonstrated in melanoma as well (30, 31). Several reports have shown that cutaneous irAEs are associated with favorable survival outcomes, including improved overall survival, progression-free survival, and objective response rate in advanced melanoma (32–35). Zhang et al. identified a strong association between cutaneous irAEs and clinical benefit in melanoma, potentially due to mechanisms such as epitope spreading and increased T-cell clonal diversity (36). Although the majority of prior studies have focused on advanced disease, emerging data also support a link between irAEs and recurrence dynamics in the adjuvant setting. A secondary analysis of the EORTC 1325/KEYNOTE-054 trial (adjuvant pembrolizumab vs placebo in stage III melanoma) demonstrated that any irAE was associated with a lower risk of recurrence or death (HR: 0.37, 95% CI: 0.24–0.57), while the development of vitiligo conferred an even more dramatic reduction in relapse risk (HR: 0.13, 95% CI: 0.02–0.95)) (16). Similarly, our findings revealed that patients who developed cutaneous irAEs within 6 months of initiating adjuvant PD-1 therapy had significantly improved RFS, a relationship that remained significant in multivariate analysis (p = 0.011). These observations were further supported by Kaplan–Meier and landmark analyses, emphasizing the prognostic relevance of cutaneous irAEs in the adjuvant immunotherapy setting.

There are plausible immunological reasons why cutaneous irAEs, in particular, correlate with improved recurrence-free survival. Checkpoint inhibitors activate T-cells broadly, and in some patients this leads to an “off-target” attack on normal melanocytes in the skin, causing rashes or vitiligo. This phenomenon may indicate that the immune system is robustly engaged against antigens shared by melanocytes and melanoma cells. Researchers have suggested that the development of skin autoimmunity reflects processes like epitope spreading and increased T-cell clonal diversity, which not only cause the skin effects but also enhance anti-tumor immunity (35). In other words, T-cells triggered by therapy may recognize a wider range of tumor-associated antigens, including some present in the skin, thereby mounting a stronger systemic attack on residual melanoma cells. Vitiligo, for example, results from immune destruction of pigment-producing cells and often points to a potent anti-melanocyte T-cell response; those same T-cells or related clones could be targeting melanoma microscopic disease (37). This hypothesis is consistent with the strikingly low relapse rates seen in patients with vitiligo. Thus, cutaneous irAEs might be a visible surrogate for effective checkpoint inhibition – an external sign that the patient’s immune system is highly active against melanoma.

In previously published studies, cutaneous irAEs have consistently been reported to occur early in the course of immunotherapy, with a median onset of approximately 4–6 weeks (38, 39). In contrast, endocrine irAEs tend to emerge much later, with a median onset of 12-14.5 weeks and a wide variability across patients (range, 1,5 to 130 weeks) (40, 41). This marked difference in timing may help explain why endocrine irAEs were not associated with improved outcomes in our cohort. Moreover, endocrine irAEs often emerge later in treatment, potentially after early relapses have occurred, introducing immortal time bias. Immunobiological differences may also contribute: while cutaneous irAEs likely reflect tumor-directed immunity, endocrine irAEs such as thyroiditis or hypophysitis may arise from broader autoimmune activity (42). Although previous studies have linked endocrine irAEs to improved outcomes, such associations were not evident in our cohort, likely due to differences in timing, frequency, and underlying mechanisms (16).

This study has several limitations. As a retrospective, single-center analysis, it is subject to inherent biases such as selection bias and unmeasured confounders. However, standardized follow-up protocols and consistent documentation of toxicity within our institution helped reduce variability in the identification of irAEs and the assessment of clinical outcomes. Although the sample size was modest, it was sufficient to detect clinically meaningful associations and is comparable to other studies in the adjuvant immunotherapy setting. Due to the limited number of patients with cutaneous irAEs and the low number of recurrence events within this subgroup, subtype-specific survival analyses (e.g., rash vs. vitiligo) could not be meaningfully performed. This remains an important area for future investigation in larger, multicenter cohorts. Additionally, the lack of external validation warrants caution, and our findings should be confirmed in larger, multicenter prospective cohorts.

Importantly, although cutaneous irAEs within the first 6 months of adjuvant anti–PD-1 therapy were associated with prolonged recurrence-free survival and a lower risk of on-treatment recurrence, these events cannot guide initial treatment decisions since they emerge only after therapy initiation. Instead, their appearance may serve as a dynamic prognostic biomarker during treatment, allowing for improved risk stratification, personalized counseling, and adaptive follow-up strategies. For example, patients with early cutaneous irAEs might benefit from less intensive surveillance and reassurance about prognosis, while those without irAEs could warrant closer monitoring. While these approaches require prospective validation, integrating irAE dynamics into real-time patient management may enhance personalized care in the adjuvant setting.

Compared to prior large-scale randomized trials such as KEYNOTE-054 and CheckMate 238, which broadly evaluated the prognostic implications of irAEs, our study offers a focused, real-world analysis of early cutaneous irAEs and their association with recurrence timing an aspect not extensively explored in previous research. While limited by its retrospective and single-center design, this dataset provides hypothesis-generating insights that may help inform future prospective studies aimed at evaluating irAEs as dynamic markers of anti–PD-1 activity. In summary, the presence of early cutaneous irAEs may not only reflect biological treatment activity but also offer clinical utility in tailoring post-treatment follow-up and counseling strategies for patients with resected high-risk melanoma.

## Data Availability

The raw data supporting the conclusions of this article will be made available by the authors, without undue reservation.
