# Supplementary material for: Association between immune-related adverse events and recurrence dynamics under adjuvant anti–PD-1 therapy in resected melanoma
Source: Front Oncol. 2025 Nov 7;15:1671315. doi: 10.3389/fonc.2025.1671315 (PMC12634362; doi:10.3389/fonc.2025.1671315)
Supplement: Supplementary file 1 [file Table1.docx]

## Table S1. Comparative Patient Characteristics by Cutaneous irAE Status

|  | Cutaneous irAE Absent n=69 | Cutaneous irAE Present n=15 | P Value |
| --- | --- | --- | --- |
| Age |  |  | 0.480 |
| <65.0 years | 44 (63.8) | 11 (73.3) |  |
| ≥65.0 years | 25 (36.2) | 4 (26.7) |  |
| Gender |  |  | 0.728 |
| Female | 31 (44.9) | 6 (40.0) |  |
| Male | 38 (55.1) | 9 (60.0) |  |
| Tumor Origin |  |  | 0.789 |
| Primary | 53 (76.8) | 12 (80.0) |  |
| Recurrence | 16 (23.2) | 3 (20.0) |  |
| Stage |  |  | 0.871 |
| Stage IIB–IIIA | 13 (18.8) | 2 (13.3) |  |
| Stage II–IIIC | 14 (20.3) | 3 (20.0) |  |
| Stage IIID–IV | 42 (60.9) | 10 (66.7) |  |
| Breslow Thickness |  |  | 0.928 |
| 1-2 mm | 7 (13.0) | 1 (9.1) |  |
| 2-4 mm | 13 (24.1) | 3 (27.3) |  |
| >4 mm | 34 (63.0) | 7 (63.6) |  |
| Tumor ulceration |  |  | 0.869 |
| Yes | 11 (20.4) | 2 (18.2) |  |
| No | 43 (79.6) | 9 (81.8) |  |
| Lymph node involvement |  |  | 0.903 |
| 0 | 13 (20.6) | 3 (25.0) |  |
| 1 | 25 (39.7) | 5 (41.7) |  |
| ≥2 | 25 (39.7) | 4 (33.3) |  |
| Melanoma Subtype |  |  | 0.800 |
| Non-acral cutaneous | 58 (84.1) | 13 (86.7) |  |
| Acral | 11 (15.9) | 2 (13.3) |  |
| Mitotisis |  |  | 0.289 |
| 1-10 | 27 (54.0) | 4 (36.4) |  |
| >10 | 23 (46.0) | 7 (63.6) |  |
| BRAF Mutation |  |  | 0.513 |
| Present | 17 (27.0) | 5 (35.7) |  |
| Absent | 46 (73.0) | 9 (64.3) |  |
| Anti-PD1 Type |  |  | 0.339 |
| Pembrolizumab | 15 (21.7) | 5 (33.3) |  |
| Nivolumab | 54 (78.3) | 10 (66.7) |  |

*Abbreviations: irAE, immune-related adverse event; PD-1, programmed cell death protein 1.*

**Table S2. Cutaneous irAE–Positive Patients: Clinical, Pathological, and Outcome**

| Patient | Sex | Age | Breslow | LN involvement | Stage | Subtype  1:Non-Acral  2:Acral | BRAF | Mitosis | Anti-PD-1 | cutaneous irAE onset (mo) | irAE Grade | Recurrence | RFS (mo) |
| --- | --- | --- | --- | --- | --- | --- | --- | --- | --- | --- | --- | --- | --- |
| P1 | Female | 69 | NE | NE | 4 | 1 | Mutant | 3 | Nivolumab | 2,8 | 1 | - | 35.4 |
| P2 | Female | 60 | 4 | 3 | 3C | 1 | Wild | 2 | Nivolumab | 3,3 | 2 | 1 | 14.6 |
| P3 | Female | 69 | 4 | 4 | 3D | 2 | Wild | 1 | Nivolumab | 2,8 | 1 | - | 30.7 |
| P4 | Female | 51 | 4 | 1 | 3C | 1 | Wild | 2 | Nivolumab | 5,4 | 1 | - | 25.2 |
| P5 | Male | 51 | NE | 3 | 3C | 1 | Wild | 3 | Nivolumab | 4,7 | 2 | - | 54.4 |
| P6 | Female | 46 | 3 | 6 | 3C | 1 | NE | 1 | Nivolumab | 5,9 | 1 | - | 22.0 |
| P7 | Male | 31 | 3 | 1 | 3B | 1 | Mutant | 1 | Nivolumab | 0,9 | 1 | - | 48.2 |
| P8 | Male | 53 | 3 | 0 | 2B | 1 | Wild | 2 | Pembrolizumab | 2,3 | 2 | - | 34.6 |
| P9 | Female | 53 | NE | NE | 4 | 1 | Wild | 3 | Nivolumab | 3,6 | 2 | 1 | 63.2 |
| P10 | Male | 63 | 4 | 1 | 3C | 1 | Wild | 2 | Nivolumab | 2,4 | 1 | - | 16.9 |
| P11 | Male | 75 | 4 | 0 | 2C | 1 | Mutant | 2 | Pembrolizumab | 3,1 | 1 | - | 21.9 |
| P12 | Male | 74 | NE | NE | 4 | 1 | Wild | 3 | Nivolumab | 4,3 | 2 | - | 15.4 |
| P13 | Male | 45 | 2 | 1 | 3A | 1 | Mutant | 2 | Pembrolizumab | 0,8 | 2 | - | 12.0 |
| P14 | Female | 64 | 4 | 1 | 3C | 1 | Mutant | 2 | Pembrolizumab | 2,7 | 2 | - | 16.7 |
| P15 | Male | 62 | 4 | 0 | 2C | 2 | Wild | 1 | Pembrolizumab | 0,5 | 2 | - | 15.4 |

*Abbreviations: LN, lymph node involvement; NE, not evaluated; irAE, immune-related adverse event; mo, months; RFS, recurrence-free survival; PD-1, programmed cell death protein 1.*
